# Proteome and phosphoproteome analysis of honeybee (*Apis mellifera*) venom collected from electrical stimulation and manual extraction of the venom gland

**DOI:** 10.1186/1471-2164-14-766

**Published:** 2013-11-07

**Authors:** Rongli Li, Lan Zhang, Yu Fang, Bin Han, Xiaoshan Lu, Tiane Zhou, Mao Feng, Jianke Li

**Affiliations:** 1 Institute of Apicultural Research/Key Laboratory of Pollinating Insect Biology, Ministry of Agriculture, Chinese Academy of Agricultural Science, Beijing 100093, China; 2 College of Bioengineering, Henan University of Technology, Zhengzhou, China

**Keywords:** Honeybee, Venom, Proteome, Phosphoproteome

## Abstract

**Background:**

Honeybee venom is a complicated defensive toxin that has a wide range of pharmacologically active compounds. Some of these compounds are useful for human therapeutics. There are two major forms of honeybee venom used in pharmacological applications: manually (or reservoir disrupting) extracted glandular venom (GV), and venom extracted through the use of electrical stimulation (ESV). A proteome comparison of these two venom forms and an understanding of the phosphorylation status of ESV, are still very limited. Here, the proteomes of GV and ESV were compared using both gel-based and gel-free proteomics approaches and the phosphoproteome of ESV was determined through the use of TiO_2_ enrichment.

**Results:**

Of the 43 proteins identified in GV, < 40% were venom toxins, and > 60% of the proteins were non-toxic proteins resulting from contamination by gland tissue damage during extraction and bee death. Of the 17 proteins identified in ESV, 14 proteins (>80%) were venom toxic proteins and most of them were found in higher abundance than in GV. Moreover, two novel proteins (dehydrogenase/reductase SDR family member 11-like and histone H2B.3-like) and three novel phosphorylation sites (icarapin (S43), phospholipase A-2 (T145), and apamin (T23)) were identified.

**Conclusions:**

Our data demonstrate that venom extracted manually is different from venom extracted using ESV, and these differences may be important in their use as pharmacological agents. ESV may be more efficient than GV as a potential pharmacological source because of its higher venom protein content, production efficiency, and without the need to kill honeybee. The three newly identified phosphorylated venom proteins in ESV may elicit a different immune response through the specific recognition of antigenic determinants. The two novel venom proteins extend our proteome coverage of honeybee venom.

## Background

Venom from social hymenoptera (wasps, bees, and ants) is used as an effective and important chemical weapon for the defense of individuals or of the colony. This can be achieved by injection of very low concentrations of venom into an enemy that reaches its bloodstream in a few minutes [1]. Honeybee (*Apis mellifera*) venom is now known to contain 12 proteins that are recognized as allergens in humans by the International Union of Immunological Societies [2]. The allergic response to these proteins in a sting victim varies from swelling, redness, pain, itching around the sting site, and potentially life-threatening allergic effects, including anaphylactic shock [3,4]. The biochemical composition of honeybee venom has been reported to have a wide array of biomolecules, such as biogenic amines, peptides and proteins [5]. In addition, honeybee venom has been well documented for pharmaceutical purposes in treatment of pathological conditions such as arthritis, neurodynia, rheumatism, skin diseases, and cancerous tumors [6-9]. Recently, it has been discovered that melittin-loaded nanoparticles can attenuate HIV-1 infectivity, demonstrating the promising potential of bee venom for treatment of HIV [10]. Currently, several pharmaceutical formulations using crude honeybee venom have been registered and are available on the European and global markets, such as Forapin, Germany; Virapin, Slovakia; Apiven, France; Melivenon, Bulgaria; and Apifor, Russia [11].

Because bee venom is a human toxin, a comprehensive characterization of its molecular composition, side effects, and toxicology is of paramount importance for the development safe pharmaceutical formulations to satisfy human demand. To further this goal, honeybee venom has been intensively examined in the past [12-14]. Due to its highly complex nature, bee venom has not been fully characterized by previous methods. Recently developed techniques, namely mass spectrometry (MS), have highly improved resolution and sensitivity, allowing the identification of thousands of proteins or peptides using a tiny amount of protein sample in a single run. A comprehensive profiling of honeybee venom has been achieved since 2005 using this robust MS platform [15-18]. Although the venom proteome of the honeybee has been significantly extended, previous studies have been performed using a singular proteomics approach such as either two dimensional electrophoresis (2-DE or gel-based proteomics) or an MS run (gel-free proteomics). Using only one technique may result in an underestimate of the proteome; 2-DE based proteomics may exclude proteins with extreme molecular weights (<15 kDa or >200 kDa) [19], while MS-based proteomics can mask the low abundance proteins [20]. However, a combination of gel-based and gel-free proteomics has proven to be an efficient protocol to increase proteome coverage of a biological sample [18,20].

At present, there are two traditional ways to collect honeybee venom: electrical stimulation (venom collected in this way is termed ESV) or manual (“reservoir disrupting”) venom extraction directly from venom glands (venom collected in this way is termed GV) [3,4]. While ESV can extract venom without the need to sacrifice any bees, GV extraction is at expense of the honeybee’s life. It is empirically recognized that GV contains more biochemical components, and it has thus been assumed it provides a more abundant potent bioactive source [3] than ESV. To address this hypothesis, the proteomes of GV and ESV were compared qualitatively and quantitatively. A better knowledge of their proteins will aid in the selection of more appropriate forms of honeybee venom to be used as a pharmacological source.

Venom toxins are secretory proteins that undergo a series of post-translational modifications (PTMs) once released from the venom glands of the honeybee, such as phosphorylation, glycosylation, and sulfation [21]. PTMs play a potential role in influencing IgE binding capacity [22] and protein immunogenicity and antigenicity [23,24]. Phosphorylation is one of the most important PTMs [25]. Phosphorylation of snake venom has the potential to alter proteolytic activity, coagulability, and neurotoxicity of proteins [26]. In honeybee venom, very few proteins, such as mellitin and icarapin, are known to be phosphorylated [18], and phosphorylation is not well characterized.

In this work we aim to better characterize honeybee venom by examining the proteomic differences of venom resulting from two different extraction techniques. By using more comprehensive techniques, we can identify previously overlooked proteins, and better understand phosphorylation patterns in honeybee venom.

## Results

### 1-DE analysis

More protein bands were detected in GV than in ESV (Additional file 1: Figure S1). A total of 22 proteins were identified in all 12 fractions, of which 21 were from GV and 13 were from ESV (Additional file 2: Table S1). Of the 22 total proteins, 12 proteins were commonly represented in the two forms of bee venom. Notably, dehydrogenase/reductase SDR family member 11-like (in GV) was identified as novel in the honeybee venom. Only five toxic proteins were found in significantly higher abundance in GV than in ESV: Api m 7, Api m 8, Api m 9, vitellogenin and PLA2-like. The other highly abundant proteins in GV were non-toxic, and were mainly associated with antioxidant activities, protein folding, and molecular transporters. In ESV, however, all 8 highly abundant proteins were toxic: Api m 3, Api m 5, Api m 6, MRJP8, MRJP9, icarapin-like, hyaluronidase, and MCDP (Additional file 3: Figure S2, panel A). The abundance of melittin, phospholipase A-2, and apamin preproprotein showed no significant differences between the two forms of venom (Additional file 2: Table S1).

### 2-DE analysis

2-DE analysis resulted in 216 and 104 protein spots (*M*r 19 kDa −88 kDa, p*I* 4.5-8.67) resolved in GV and ESV, respectively (Additional file 4: Figure S3). Of these, 70 of the 216 spots in GV and 24 of the 104 protein spots in ESV altered their abundance (> 1.5-fold, *p* < 0.05). Among those which showed a greater than 1.5-fold abundance change, 53 of the 70 GV spots and 17 of the 24 ESV spots were successfully identified corresponding to 24 non-redundant proteins in GV and ESV (Additional file 5: Table S2). Five non-redundant proteins were shared in the two forms of bee venom (Additional file 4: Figure S3, red arrow), and 19 other proteins were specifically expressed in GV (Additional file 4: Figure S3, blue arrow), but none of these proteins were expressed only in ESV. In ESV, 4 venom toxins had a higher abundance (dipeptidyl peptidase IV precursor (Api m 5), venom allergen acid phosphatase (Api m 3), icarapin-like, and phospholipase A-2 (PLA2) (Additional file 3: Figure S2, panel B). In GV, however, only two toxic proteins were abundant: venom serine carboxypeptidase (Api m 7) and arginine kinase. The other 17 specifically detected proteins in GV were involved in antioxidant systems, protein folding and molecular transporters, carbohydrate or energy metabolism (Additional file 3: Figure S2, panel B). The remaining unidentified differential protein spots could be attributed to either their low abundance to produce enough spectra or that the search scores in the databases did not produce unambiguous results.

### Shotgun analysis

Twenty proteins were identified in the shotgun analysis, of which 14 proteins were found in both GV and ESV. Five additional proteins were identified only in GV, and one protein was identified only in ESV (Additional file 6: Table S3). Noticeably, secapin, proactivator polypeptide, danJ homolog subfamily B member 11-like and histone H2B.3-like were specifically identified in the shotgun approach.

Abundance quantification showed that 9 venom toxins had higher abundance in ESV than in GV: MCDP, PLA2, Api m 5, MRJP8 and 9, Api m 3, hyaluronidase, icarapin-like, Api m 6 and LOC408666. Vitellogenin (Api m 12) had higher abundance in GV than in ESV (Additional file 3: Figure S2, panel C). Five others (melittin, secapin, PLA2-like, proactivator polypeptide isoform 1, dnaJ homolog subfamily B member 11-like) showed no significant difference between GV and ESV (Additional file 6: Table S3). Similar to the above 1-DE and 2-DE analysis, proteins identified in GV were also associated with antioxidant activities, protein folding and molecular transporters (Additional file 3: Figure S2, panel C).

**Figure 1:**
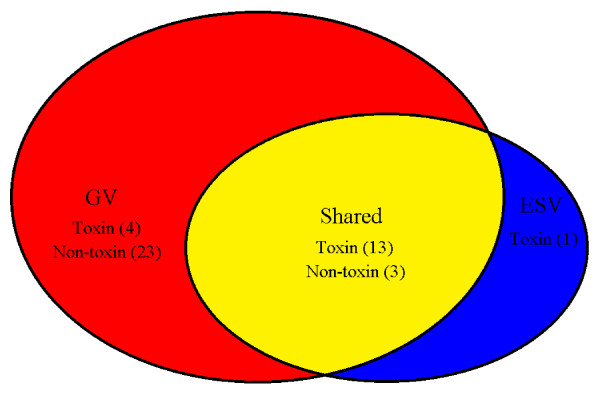
**Number comparison of the identified protein in venom of *A. m. ligustica *manually collected from venom glands (GV) and electrically stimulated (ESV).** Venn diagram shows the taxonomical distribution of the identified 44 proteins. Numbers in the parenthesis indicate the amount of proteins ascribed to venom toxins and non-toxins, respectively.

In total, 44 non-redundant proteins were identified in the two forms of bee venom, 16 proteins were shared in GV and ESV: 13 venom toxins and 3 non-toxins. In GV, 27 proteins were specifically expressed: 4 toxins and 23 non-toxins. In ESV, only toxin MCD peptide was specifically expressed. Generally, 17 proteins were identified in ESV and 43 proteins were identified in GV, respectively (Table 1 and Figure 1).

**Table 1:** Classification of identified proteins in honeybee venom manually extracted from venom gland and electrical stimulation by gel-based and gel-free techniques

**Protein accession no**	**Protein name**	**Functional category**	**Secretory protein**	**Source**	**Venom manually extracted from venom gland**			**Venom extracted from electrical stimulation**		
					**1-D E**	**2-DE**	**Gel-free**	**1-DE**	**2-DE**	**Gel-free**
gi|5627	Phospholipase A-2 (Api m 1)	Bee venom toxins	Y	2, 57	√	√	√	√	√	√
gi|58585182	Hyaluronidase precursor (Api m 2)		Y	2, 57	√		√	√		√
gi|66821891	Venom allergen acid phosphatase (Api m 3)		N	2	√	√	√	√	√	√
gi|28201825	Melittin (Api m 4)		Y	2, 57	√		√	√		√
gi|187281543	Venom dipeptidyl peptidase IV precursor (Api m 5)		Y	2, 57	√	√	√	√	√	√
gi|94400907	Allergen Api m 6 precursor (Api m 6)		Y	2, 57	√		√	√		√
gi|187281550	Venom carboxylesterase-6 precursor (Api m 8)		Y	2, 57	√			√		
gi|60115688	Icarapin-like precursor (Api m 10)		Y	2, 57	√	√	√	√	√	√
gi|67010041	Major royal jelly protein 9 precursor (Api m 11)		Y	2, 57	√		√	√		√
gi|58585070	Major royal jelly protein 8 precursor (Api m 11)		Y	2, 57	√		√	√		√
gi|58585166	Apamin preproprotein		Y	2, 57	√			√		
gi|223850	Secapin		N	5,38			√			√
gi|110758297	Phospholipase A2-like		Y	2, 57	√		√	√		√
gi|1708948	Mast cell degranulating peptide		Y	2, 57				√		√
gi|58585116	Venom serine protease 34 precursor (Api m 7)		Y	2, 57	√	√				
gi|226533687	Venom serine protease 34 precursor Venom serine carboxypeptidase precursor (Api m 9)		Y	2, 57	√					
gi|58585146	Arginine kinase		N	36		√				
gi|58585104	Vitellogenin (Api m 12)		Y	57	√		√			
gi|380020933	Glutathione S-transferase-like isoform 1 (GstS1)	Antioxidant systems	N	57		√				
gi|58585086	Transferrin 1 precursor (Tsf1)		Y	57		√				
gi|110755367	Toll-like receptor 13-like isoform 1 (TLRs)		Y	57		√				
gi|66514614	Chitinase-like protein Idgf4-like		Y	57		√				
gi|283436152	Peroxiredoxin-like protein (Prx)		N	57		√				
gi|295849268	Superoxide dismutase 1 (Sod1)		N	57		√				
gi|22982210	Heat shock protein cognate 4 (Hsc)		N	57		√				
gi|149939403	Hexamerin		N	57	√	√				
gi|328790510	DnaJ homolog subfamily B member 11-like	Protein folding and molecular transporters	Y	57			√			√
gi|335892796	Peptidyl-prolyl cis-trans isomerase B precursor		Y	57	√					
gi|328782499	Proactivator polypeptide isoform 1		Y	57			√			√
gi|328780884	Apolipophorins isoform 1		N	57	√		√			
gi|328780886	Apolipophorins-like		Y	57	√					
gi|110749558	Histone H2B.3-like		N	57			√			
gi|66535784	Odorant binding protein 21 precursor (Obp 21)		Y	57		√				
gi|66515272	V-type proton ATPase catalytic subunit A-like (V-ATPase subunit A)	Carbohydrate and energy metabolism	N	57		√				
gi|328785025	ATP synthase subunit beta, mitochondrial (ATPsyn-beta)		N	57		√				
gi|328776580	Enolase-like (Eno)		N	57		√				
gi|66525576	Aldose reductase-like (AR)		N	57		√				
gi|328780312	Alcohol dehydrogenase [NADP+] A-like (Adh)		N	57		√				
gi|66506786	Malate dehydrogenase cytoplasmic-like (MDH1)		N	57		√				
gi|66550890	Phosphoglycerate mutase 2-like (Pglym)		N	57		√				
gi|328779578	Lysozyme c-1	Others 4	Y	57	√		√			
gi|328783193	Dehydrogenase/reductase SDR family member 11-like (SDR )		N	57	√		√			
gi|328789531	Hypothetical protein LOC408666		Y	57		√	√		√	√
gi|48095525	Tubulin beta-1 chain		N	57		√				

All proteins are identified as *Apis mellifera* origin. Accession number is the unique number given to mark the entry of a protein in the database of NCBInr used to search against in Mascot software. √ indicates the proteins identified by corresponding proteomics approaches such as 1-DE, 2-DE and shotgun. Y and N indicate whether a protein is a secretory protein or not, which the evidence is given in source column.

**Figure 2:**
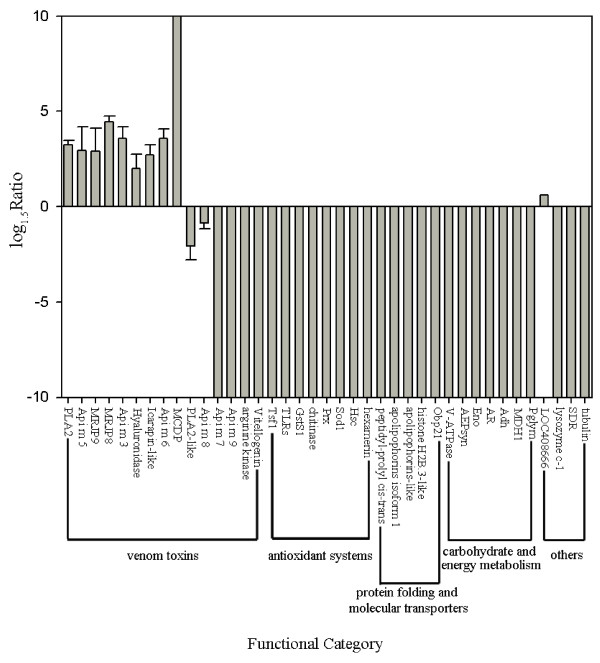
**Quantitative comparisons of differentially abundant proteins in honeybee (*A. m. ligustica) *venom manually collected from the venom gland (GV) and electrically stimulated (ESV).** The ratio of the protein abundance is ESV to GV. The positive values indicate higher protein abundance in ESV, negative values denote higher protein abundance in GV. The ratio is limited to 10, and error bar is standard deviation.

Of the 39 differentially expressed proteins between GV and ESV (Figure 2), 29 were more highly expressed in GV: 6 venom toxins and 23 other non-toxin proteins associated with antioxidant systems, carbohydrate or energy metabolism, protein folding and molecular transporters. In contrast, 10 proteins were more highly expressed in ESV than GV: 9 venom toxins, and one non-toxin related protein. The three other venom toxins such as melittin, secapin, apamin had no significant difference between GV and ESV.

### Phosphopeptide analysis

**Figure 3:**
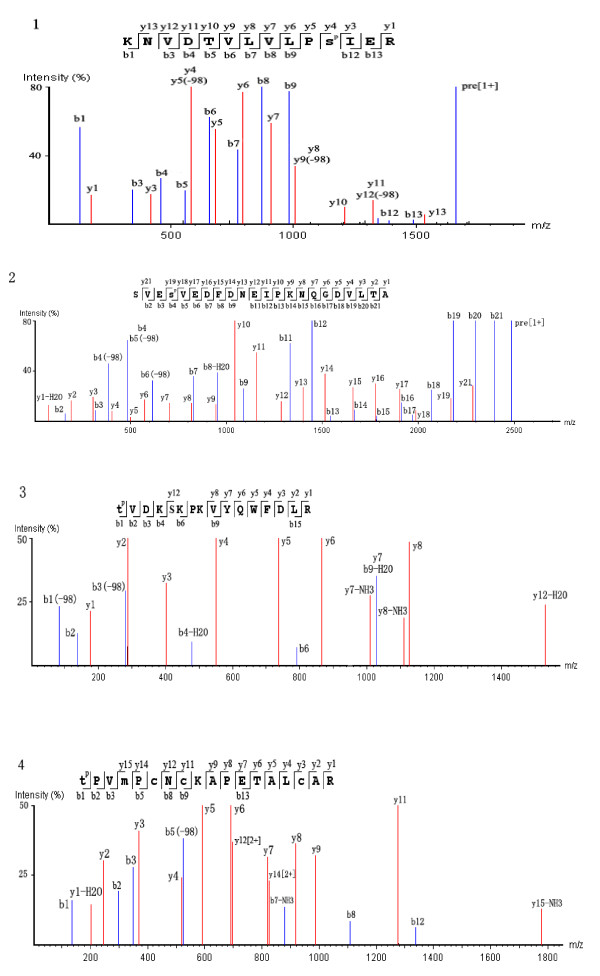
**Tandem mass spectra of phosphorylated peptides from electrically stimulated venom *of A. m. *ligustica.** The blue and red color codes represent ions of b and y, respectively. The precursor ion marked as pre. Panel **1** and **2** are spectra of KNVDTVLVLPS(+79.97)IER phosphorylated on S43 and SVES(+79.97)VEDFDNEIPKNQGDVLTA phosphorylated on S205 in icarapin-like precursor. Panel **3** is a spectrum of T(+79.97)VDKSKPKVYQWFDLR that phosphorylated on T145 in phospholipase A-2. Panle **4** is spectrum of T(+79.97)PVMPCNCKAPETALCAR that phosphorylated on T23 in apamin preproprotein.

ESV contained 82% of venom toxins based on the above analysis, and was, therefore used for the phosphorylation analysis of honeybee venom. Currently, a total of 4 phosphopeptides corresponding to three venom toxins have been identified (Table 2 and Figure 3): icarapin-like precursor was phosphorylated at S43 and S205; phospholipase A-2 was phosphorylated at T145 and apamin preproprotein was phosphorylated at T23.

**Table 2:** Identification of phosphorylated sites on peptides in honeybee venom collected by electrical stimulation

**Protein name**	**Mass**	**−10lgP**	**Phosphorylation site**	**No. of spectra**	**Z**	Site*	**Modified peptides and sites**
Icarapin-like precursor, 33-46	1661.8752	39.63	S43	2	2	33-46	R.KNVDTVLVLPS(+79.97)IER.D
Icarapin-like precursor, 202-223	2485.0896	56.65	S205	2	2	202-223	R.SVES(+79.97)VEDFDNEIPKNQGDVLTA
Phospholipase A-2, 145-160	2169.0061	44.56	T145	1	4	145-160	Y.T(+79.97)VDKSKPKVYQWFDLR.K
Apamin preproprotein 23-40	2170.9033	30.85	T23	2	4	23-40	V.T(+79.97)PVMPCNCKAPETALCAR.R

Protein name and mass of the identified proteins searched against the NCBInr database in 2.5. Modified site is the position of the initial and final amino acids of peptide in the protein sequence. Phosphorylated amino acid is labeled with S^p^. Site* is the position of the initial and final amino acids of the peptide in the protein sequence. No. of spectra means the number of the identified phosphopeptides’s mass spectra. Z means the charge of identified phosphopeptide. The peptides are accepted when -10lgP > 20 (p < 0.01) as well as FDR <0.1%.

## Discussion

We used complementary proteomics approaches to gain insight into the proteome differences of honey bee venom extracted using two different methods (GV and ESV) and to map the phosphorylation sites of the most common used form of venom for therapeutic purposes. Of the 44 proteins identified in both GV and ESV, most of venom toxins were in higher abundance in ESV than in GV. The most abundant proteins detected in GV were mainly involved in protection of the venom gland from tissue damage or in supporting glandular development. This suggests that electrical stimulation could generate a more pure venom as its contamination with cellular proteins is much smaller than that of the manual method. This method is also convenient for commercial production and does not sacrifice the bees in the collection process. This is in sharp contrast with the manual milking of bee venom (GV), in which the bee is sacrificed, and the venom is contaminated by non-toxin proteins that may leak from the gland tissue that is cut/disrupted during venom collection. This provides a new scientific basis for the use of ESV as a potential pharmacological source over GV, taking into consideration both production efficiency and honeybee welfare. Four novel phosphorylation sites identified in three venom proteins may be involved in specific elicitation of an immune response via recognition of antigenic determinants [18]. The identification of two novel venom proteins significantly extends our in-depth understanding of the biological nature of honeybee venom.

More recently, Resende et al. (2013) [18] has used a higher resolution of MS than our adopted MS for the profiling of bee venom, and identified many new proteins and three phosphorylation sites mapped to icarapin and melittin. Using our gel-based and gel-free proteomics approaches, two novel proteins, SDR and histone H2B.3-like, were identified, and phosphorylation of phospholipase A-2, apamin preproprotein and S43 in icarapin-like precursor were assigned as novel sites. This indicates that the combined gel-based and gel-free proteomics approaches are a robust protocol for the achievable identification of proteome and phosphoproteome of bee venom.

In order to effectively utilize venom in defense of their community, the honeybee using its venom inflicts the invader with the utmost suffering through systemic IgE-mediated allergic reactions after insect stings. This could result in potentially life-threatening and sometimes fatal immune-mediated anaphylaxis. Our study has identified all the 12 allergens (Api m 1–12) in the honeybee venom. Melittin (Api m 4) is the major allergen responsible for intense local pain [27] and can trigger the lysis of a wide range of cells [28]. Phospholipase A-2 (Api m 1) is important for the specific IgE induction in sting victims [9]. Hyaluronidase (Api m 2) facilitates the diffusion of other venom constituents through the interstitial space [29]. Api m 3, 5–12 are recognized as a strong IgE and T-cell response to bee venom of the sting victim [30]. Except for Api m 7–9 and melittin, the higher abundance of all above allergens in ESV are thought to be a survival strategy of the honeybee to effectively defend individuals and the colonies they live in [31].

In addition, the identified other toxins, such as MCD peptide, venom serine carboxypeptidases, venom serine proteases, arginine kinase, apamin, and secapin, work as enhancers for a massive release of histamine [32], degradation of insect neurotransmitters [33], mediation of immunity-related processes [34], phosphorylation of venom proteins [16,35-37], and influencing the nervous system as a neurotoxic polypeptide [38].

Although honeybee venom is mainly physiologically effective for toxicological suffering in the sting victim, the reported pharmacological applications are mainly from the toxins. Melittin and phospholipase A-2 have been reported to inhibit tumor cell proliferation and thus have potential value for the cure of cancer [39,40], and the attenuation of HIV-1 infectivity [10]. Hyaluronidase can be used as an anesthetic, analgesic, anti-cancer agent and also to help the spread of drugs in tissues [41]. Dipeptidyl peptidase IV has functionally suppressed peritoneal dissemination and progression of ovarian carcinoma and inhibits malignant phenotypes of prostate cancer cells [42,43]. The MCD peptide has reported anti-inflammatory activity at higher concentrations, which can be used as a tool in studying secretory mechanisms of mast cells, basophils, and leukocytes [44]. Most toxin proteins had either a higher abundance in ESV (phospholipase A-2, hyaluronidase, the MCD peptide, and venom dipeptidyl peptidase IV) or were found in equal abundance in GV and ESV (melittin, apamin and secapin). Therefore, the utilization of ESV as a potential pharmacological source is a more efficient and convenient choice as a valuable source for the future development of novel human therapeutics than that of GV [45].

In GV, many non-venom toxic proteins were identified even though manually milking of the venom from the gland was performed carefully. The toxin component in the venom of manual collection has significantly lower content than venom from a honeybee’s natural sting. These non-venom toxic proteins were may mainly contaminated by leaking when gland tissues were damaged. They are involved in many pathways for the normal functionality of venom glands to secrete venom toxins and development, such as antioxidant systems, protein folding, molecular transporters, carbohydrate and energy metabolism. They might play an important role in protection of the secretory cells of the venom gland from the harmful damage of toxins [16]. Although some of the proteins were identified as putative secretory proteins, we could not rule out that they are the remaining trace amounts of bee venom toxin, since the reported toxin accounts for >80% of venom content [5,18,38], this requires further study to confirm if they are real toxin proteins in bee venom.

Phosphorylation is a quite rapid biological reaction in quick response to external stimulus and internal signals. The phosphorylation site is recognized as an important allergenic epitope, and the allergenicity of the proteins is affected by a change in the epitope [46,47]. To fully exert the toxic power towards protecting the honeybee colony, the phosphorylation of venom toxin is probably to enhance the efficiency of local tissue damage, induction of death in other insects and pain, and allergy and inflammation in higher organisms [4,48]. To this effect, icarapin-like protein is suspected in evoking an immune response after a bee sting through protein phosphorylation [49]. Phosphorylation of phospholipase A-2 may be responsible for increasing its lysis activity [50]. Apamin is an important neurotoxic polypeptide, and its phosphorylation may participate in the modulation of Ca^2+^-activated K^+^channel [51,52] that acts on the central nervous system [53]. The mapped S205 in Icarapin is the same as the report of Ferreira Resende (2013) [18]. The other assigned sites in phospholipase A-2 and Apamin and S43 in icarapin-like precursor are novel.

## Conclusions

Using complementary gel-based and gel-free proteomic techniques, we have found that proteins in ESV are mainly composed of toxin proteins and most of them are highly abundant. Proteins identified in GV are mainly those which have been contaminated by the venom tissue damage during the milking process. ESV is the better form to more efficiently utilize honeybee venom as a potential pharmacological source, in terms of obtaining it commercially and without the need to sacrifice each individual bee. The identification of two novel proteins and three phosphorylation sites on venom proteins provides key information to gain new insight into the biochemical properties of honeybee venom.

## Methods

### Chemical reagents

The chemicals used for 1-DE, 2-DE and Formic acid (FA) were purchased from Sigma (St. Louis, MO, USA) except for Biolyte and immobilized pH gradient (IPG) strips that were purchased from Bio-Rad (Hercules, CA, USA). Dithiothreitol (DTT) and iodoacetamide (IAA) were from Merck (Darmstadt, Germany). The modified sequencing grade trypsin was from Roche (Mississauga, ON, Canada). Ti^4+^-IMAC materials were kindly offered by Dalian Institute of Chemical Physics. Trifluoroacetic acid (TFA) and acetonitrile were from J.T.Baker (Phillipsburg, NJ). Chemicals used for silver-staining were purchased from Beijing Shiji Co. (Beijing, China).

### Venom collection

The venom samples were collected from five colonies of Italian honeybees (*Apis mellifera ligustica*) in the apiary of The Institute of Apicultural Research, Chinese Academy of Agricultural Sciences in Beijing. For the GV harvested from honeybee workers, manual (or reservoir disrupting) venom extraction was performed as follows: 100 guard bees were captured near the entrance of the colony and anesthetized by chilling at −20°C. Afterwards, each individual was dissected, the sting apparatus and the venom reservoirs were removed, and a filter paper blot was used to prevent contamination by any hemolymph that had stuck onto the outside. A capillary tube was used to pierce the reservoir to collect the venom from the gland followed by mixing with lysis buffer (8 M urea, 2 M thiourea, 4% CHAPS, 20 mM Tris-base, 30 mM DTT, proteinase inhibitor cocktail) for immediate protein extraction. The venom collected using the ESV method was collected by a device with a mild electric shock through wires above the collecting tray. The beehive lid and the cover cloth were taken off and then a tray (sprayed with the proteinase and phosphatase inhibitor cocktail before transfer to the hives) was put on the hive top and it was assessed that the steel wires were facing down. The wires were alternately charged to a maximum of 3 volts. When shocked, the bees stung the nylon taffeta under which was a glass collection plate. The venom dried rapidly on glass plates and was scraped off with a knife. The collected venom was kept at −80°C until use. Three independent biological replicates were produced for the protein abundance quantitation in 1-DE, 2-De, and shotgun analyses. Protein preparation was performed as described previously [54]. To do comparable quantitation between the two forms of venom, the content of extracted protein was determined [54] and equal amounts of protein sample were subjected to 1-DE, 2-DE, and shotgun analyses. The protein sample was divided into three parts for the following analysis.

### One-Dimensional Gel Electrophoresis (1-DE)

1-DE was performed by the previously described protocol [55]. The first part of the venom protein sample (6.7 μg/1 μl buffer) of GV and ESV was dissolved in the above mentioned lysis buffer, 50 μg of protein sample (7.5 μl) were mixed with a loading buffer (2.5 μl) [62 mM Tris (pH 6.8), 10% (v/v) glycerol, 2.5% (w/v) SDS, and 5% (v/v) 2-mercaptoethanol, pH 6.8] for the subsequent separation. The gel was stained by MS compatible silver-staining method according to Han et al. [55]. The entire lanes were cut into 12 fractions and transferred to sterile microcentrifuge tubes, then washed and subjected to in-gel tryptic digestion according to Han et al. [55]. Briefly, The silver-stained gel slices were excised and destained in a 200 μl solution (30 mmol/L K_3_Fe(CN)_6_: 100 mmol/L Na_2_S_2_O_3_ = 1:1) until the gel spots were transparent, then dried for 10 min with ACN (100%). They were then further dried for 30 min using a Speed-Vac system (RVC 2–18, Marin Christ, Germany). The gel slices were reduced with 10 mM DTT for 1 hour and alkylated with 50 mM iodoacetamide for 1 h in the dark. Next, the trypsin solution (10 ng/μl) was added to the dried gel slices then the samples were incubated for 14 h at 37°C. 5% TFA and 50% acetonitrile acid was used to extract the peptide fragments from the tryptic digests. Finally, the supernatant of each gel slice was concentrated to 20 μl using a Speed-Vac system for the subsequent MS analysis. Three replications were performed for each gel lanes.

### Two-Dimensional Gel Electrophoresis (2-DE)

2-DE was performed by a previously described protocol [56]. 500 μg of the second part of the protein sample of GV and ESV were dissolved in the lysis buffer, and then the mixture (1 μl mixture/4 μl rehydration buffer) was added into a rehydration buffer (8 M uera, 2% CHAPS, 0.001% bromophenol blue, 45 mM DTT, and 0.5% Biolyte, pH 3–10) for the 2-DE runs. The gel staining was accomplished by the same method as the 1-DE. Three independent and reproducible 2-DE gel images from the two venom samples were digitized with Image Scanner III (GE Healthcare, Piscataway, NJ, USA), and the gel images were analyzed with Progenesis SameSpot (Version 4, Nonlinear Dynamic, UK) software. All gels were matched with one of the selected reference gels. The following gel, protein spot calculation, and normalization were done as previously described [56]. Protein abundance was represented as the means and standard deviations from the triplicate experiments, and the statistical significance between the two means was analyzed by one-way ANOVA (Samespot, version 4, nonlinear Dynamics, UK) using a q-value multiple test. Only spots statistically significant with at least 2-fold changes and p < 0.05 were considered to be differential protein spots. The q-value was used to estimate false positive results that determine adjusted p-values for each test.

### MS/MS analysis

In-gel digestion was done following a previously published protocol [55]. The digested protein spots were analyzed by the LC-MS/MS system (QTOF G6520, Agilent Technologies, Santa Clara, CA), equipped with a microwell-plate autosampler G1377D (maintained at 4°C), a capillary sample loading pump G1382A, a nano pump G2225A, and a HPLC-Chip interface (Chip Cube G4240A). The LC-chip (Agilent Technologies) used for analysis of 1-DE gel slices was constituted of a Zorbax 300SB-C18 enrichment column (160 nL, 5 μm) and a Zorbax 300SB-C18 analytical column (75 μm × 150 mm, 5 μm) (G4240-62010, Agilent Technologies, Santa Clara, CA). Elution from the analytical column was performed by a binary solvent mixture composed of water with 0.1% formic acid (solvent A) and acetonitrile with 0.1% formic acid (solvent B). The following gradient program was used: 3% to 8% solvent B for 1 min, 8% to 40% B for 79 min, 40% to 85% B for 30 min, and 85% B for 2 min. The LC-chip used for analysis of the 2-DE protein spots was the same as that used for1-DE, but the enrichment column was 40 nL. The following gradient program was used: 3% to 8% solvent B for 1 min, 8% to 40% B for 5 min, 40% to 85% B for 1 min, and 85% B for 1 min. The mass spectrometric conditions of Han et al. were followed [55].

### Shotgun analysis

50 μg of the third part of GV and ESV samples was digested in-solution as described by Han et al. [55]. Before the MS/MS run, a digested protein sample was separated using a PL-SCX (50 × 0.3 mm, 1000 Å, Agilent Technologies, Santa Clara, CA) exchange media online. The peptides were separated into seven fractions with salt step of 5, 10, 20, 30, 50, 70, and 90 mM of NH_4_HCO_3_ for the gradient elution. Each fraction was analyzed online with LC-MS/MS system (QTOF G6520, Agilent Technologies, Santa Clara, CA) using the same mass spectrometric parameters as 1-DE, except the loading flow rate was 0.3 μl/min, and the chip flow rate was 200 nl/min. The following gradient program was used: 3% to 3% solvent B for 5 min, 3% to 4% B for 2 min, 4% to 5% B for 3 min, from 5% to 10% B for 5 min, 10% to 12% B for 5 min, maintained 12% B for 5 min, 12% to 13% B for 5 min, maintained 13% B for 5 min, 13% to 20% B for 5 min, 20% to 50% B for 5 min, maintained 50% B for 5 min, from 50% to 85% B for 5 min, and 85% B for 5 min (the concentration of solvent B (acetonitrile with 0.1% formic acid) was achieved by mixing it with solvent A: water with 0.1% formic acid.

### Protein identification

Tandem mass spectra were retrieved using MassHunter software (version B.04, Agilent Technologies). The combined mgf file of MS/MS data was generated by Distiller (version 2.4, Matrix Science, UK) and searched against a sequence database generated from the NCBInr protein sequences of *Apis mellifera* (downloaded April, 2012, version 4.5 of the honeybee genome) with a total of 72,672 sequences, expanded with sequences of *Drosophila melanogaster* (downloaded April, 2012), *Sacharomyces cerevisiae* (downloaded April, 2012), and a common repository of adventitious proteins (cRAP, from The Global Proteome Machine Organization, downloaded April, 2012). MS/MS data were searched using in-house Mascot (version 2.4.0, Matrix Science, UK). Carbamidomethyl (C) and Oxidation (M) were selected as fixed and variable modifications, respectively. Enzyme, trypsin; missed cleavages, 1; peptide tolerance, 50 ppm; MS/MS tolerance, 0.05 Da. The false discovery rate (FDR) for the shotgun and 1-DE analyses was set at <1% when the decoy (reverse) sequences were included. Protein identification was accepted if they had >95% confidence and contained at least two unique peptides. If a protein was identified on the basis of one peptide for the shotgun and 1-DE analyses, protein identification having theoretical probability of obtaining a false positive was set at <1 in 1000, meaning that these peptides must have a (false positive) Mascot expect value of ≤ 0.001. The presence of an N-terminal secretion signal peptides of the identified proteins was verified using the SignalP 4.1 Server (http://www.cbs.dtu.dk/services/SignalP/) [57]. The D-cutoff for signal-TM networks was set to 0.35.

### Quantification of protein abundance

The protein abundance identified by 1-DE and shotgun analyses was estimated by the exponentially modified Protein Abundance Index (emPAI) [58], which was automatically calculated by the MASCOT search engine. The emPAI can be directly used for reporting approximate protein abundance in a large-scale analysis [59]. The comparative analysis of the protein abundance was performed using the one-way ANOVA (SPSS statistics 17.0, version 17.0.0, IBM) and Duncan’s multiple tests. An error probability of *p* < 0.05 was considered statistically significant.

### Phosphopeptide enrichment using Immobilized Metal Affinity Column (IMAC)

Ti^4+^-IMAC material was prepared as described by Zhou et.al [60]. Phosphopeptide enrichment using IMAC was followed by Yu et.al with minor modification [61]. 100 μl of 500 mM K_2_HPO_4_ was used to elute phosphopeptide from Ti^4+^-IMAC using 30 min of agitation and 15 min of sonication and finally centrifugation at 15000 g for 5 min. The two rounds of supernatant were collected as enriched phosphopeptides and lyophilized for further analysis.

The dissolved phosphopeptides in 35 μl of 0.1% FA were subjected to MS analysis according the above shotgun analysis without using SCX pre-fraction.

The phosphopeptide MS data were searched against PEAKS studio (version 6.0, Bioinformatics solutions Inc., Canada) and searched against the database in the same manner as the above shotgun analysis with the following parameters: carbamidomethylation (C) was selected as the fixed and oxidation; (M) and phosphorylation (STY) were selected as the variable modifications; taxonomy, all entries; enzyme, trypsin; missed cleavages, 2; peptide tolerance, ± 50 ppm; and MS/MS tolerance, ± 0.05 Da. A fusion-decoy strategy was employed to control FDR of protein, and peptide identification used the cutoff score of >20 (−10 lgP) and FDR < 0.1%. Similar to unmodified peptides, incorrect peptide spectrum matches (PSMs) of modified peptides have an equal chance of being derived from either the target or decoy database. The target/decoy database approach is used to estimate the false discovery rate for modified peptide identification. The success of two consecutive b-or y-type site-determining ions was required to assign a confident site [62].

### Animal ethical use issues

Honey bees are not a regulated invertebrate. Therefore, no ethical use approval is necessary.

## Competing interests

The authors declare that they have no competing interests.

## Authors’ contributions

LRL, ZL and LJK conceived of this study, and participated in its design and coordination. LRL carried out the experiments. FY, ZTE and FM contributed to the sample collection and MS analysis. HB and LXS participated in the enrichment of phosphopeptide and shotgun analysis. LRL, ZL and LJK performed the data analysis and drafted the manuscript. All authors read and approved the final manuscript.

## Supplementary Material

Additional file 1: Figure S1 Separation of honeybee (*A. m. ligustica*) venoms manually extracted from the venom gland (GV) and electrical stimulation (ESV) using one-dimensional gel electrophoresis. 50 μg of protein sample are subjected to each gel lane with three replications in each sample. The proteins are separated into 12 fractions (marked by red boxes and labeled 1-12) and stained using a mass spectrometry compatible silver-staining method. The molecular weight markers (M) are indicated on the left. Proteins are identified by high-performance liquid chromatography chip quadruple time-of-flight tandem mass spectrometry as described in 2.3.3. Click here for file

Additional file 2: Table S1 Identification and quantitation of proteins in honeybee venom manually extracted from venom gland (GV) and electrical stimulation (ESV) by 1-DE analysis. Click here for file

Additional file 3: Figure S2 Quantitative comparisons of differential abundant proteins in honeybee (*A. m. ligustica)* venom manually collected from venom glands (GV) and electrical stimulated (ESV). The ratio of the protein abundance is ESV to GV. The positive values indicate higher protein abundance in ESV, negative values denote higher protein abundance in GV. The ratio is limited to 10, and error bar is standard deviation. Panel A, B and C are comparison of protein abundance analyzed by one-dimensional gel electrophoresis (1-DE), two-dimensional gel electrophoresis (2-DE) and shotgun analysis, respectively. Click here for file

Additional file 4: Figure S3 Separation of honeybee (*A. m. ligustica*) venoms manually extracted from the venom gland (GV) and electrical stimulation (ESV) using two-dimensional gel electrophoresis (2-DE). 500 μg of each sample are subjected to 2-DE and the proteins are stained using a mass spectrometry compatible silver-staining method. Number-labeled spots are cut out and subjected to tryptic digestion for mass spectrometry analysis. Click here for file

Additional file 5: Table S2 Identification of different abundant proteins between honeybee venom manually extracted from venom glands (GV) and electrical stimulation (ESV) by 2-DE analysis. Click here for file

Additional file 6: Table S3 Identification and quantitation of protein in honeybee venom manually extracted from venom gland (GV) and electrical stimulation (ESV) by shotgun analysis. Click here for file
