# Supplementary material for: Proteome and phosphoproteome analysis of honeybee (Apis mellifera) venom collected from electrical stimulation and manual extraction of the venom gland
Source: BMC Genomics. 2013 Nov 7;14:766. doi: 10.1186/1471-2164-14-766 (PMC3835400; doi:10.1186/1471-2164-14-766)
Supplement: Additional file 1: Figure S1 — Separation of honeybee (A. m. ligustica) venoms manually extracted from the venom gland (GV) and electrical stimulation (ESV) using one-dimensional gel electrophoresis. 50 μg of protein sample are subjected to each gel lane with three replications in each sample. The proteins are separated into 12 fractions (marked by red boxes and labeled 1-12) and stained using a mass spectrometry compatible silver-staining method. The molecular weight markers (M) are indicated on the left. Proteins are identified by high-performance liquid chromatography chip quadruple time-of-flight tandem mass spectrometry as described in 2.3.3. [file 1471-2164-14-766-S1.doc]

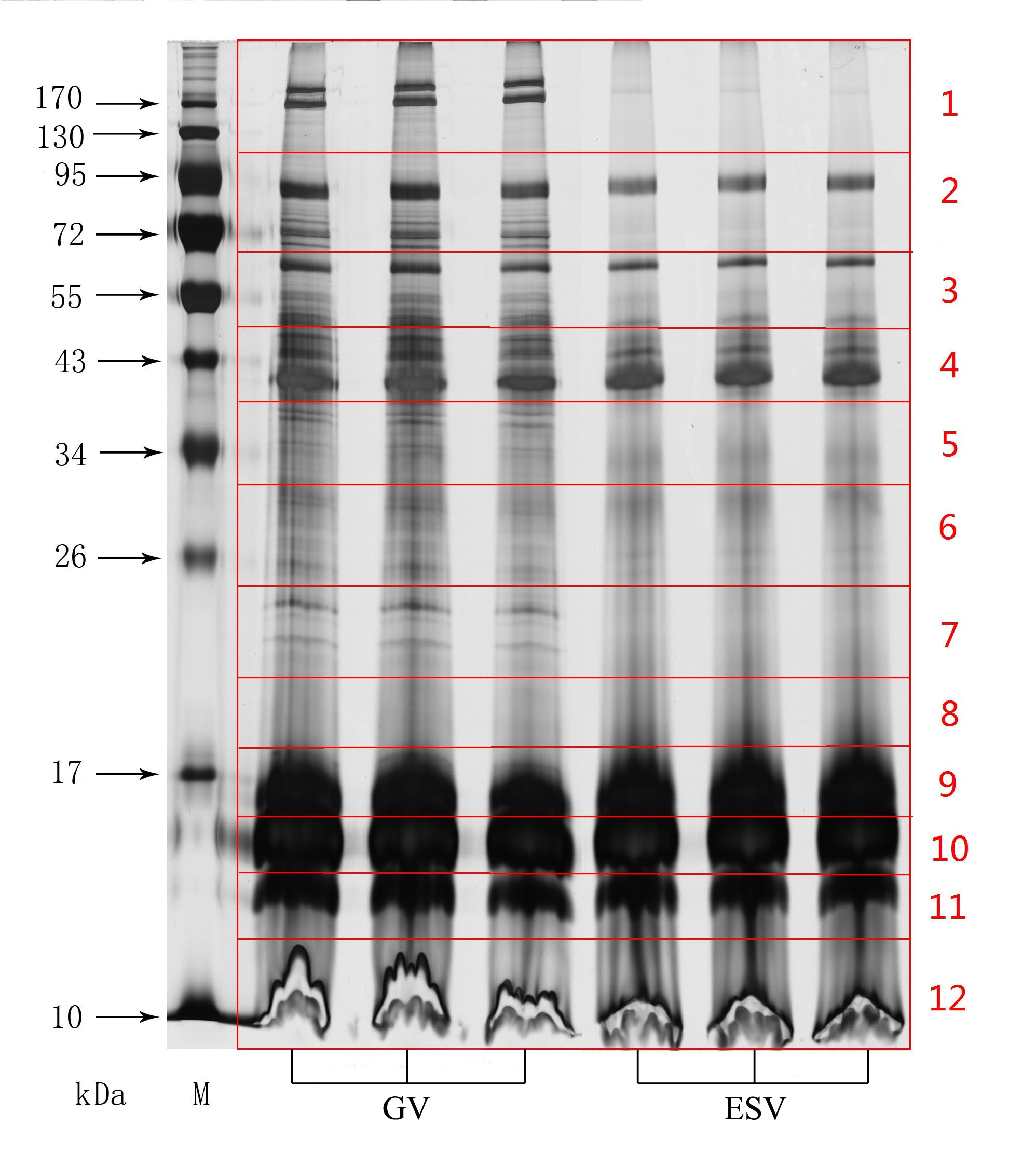


**Additional file 1: Figure S1.** Separation of honeybee (*A. m. ligustica*) venoms manually extracted from the venom gland (GV) and electrical stimulation (ESV) using one-dimensional gel electrophoresis.50 μg of protein sample are subjected to each gel lane with three replications in each sample. The proteins are separated into 12 fractions (marked by red boxes and labeled 1-12) and stained using a mass spectrometry compatible silver-staining method. The molecular weight markers (M) are indicated on the left. Proteins are identified by high-performance liquid chromatography chip quadruple time-of-flight tandem mass spectrometry as described in 2.3.3.
