# Supplementary material for: Proteome and phosphoproteome analysis of honeybee (Apis mellifera) venom collected from electrical stimulation and manual extraction of the venom gland
Source: BMC Genomics. 2013 Nov 7;14:766. doi: 10.1186/1471-2164-14-766 (PMC3835400; doi:10.1186/1471-2164-14-766)
Supplement: Additional file 2: Table S1 — Identification and quantitation of proteins in honeybee venom manually extracted from venom gland (GV) and electrical stimulation (ESV) by 1-DE analysis. [file 1471-2164-14-766-S2.doc]

**Additional file 2: Table S1. Identification and quantitation of proteins in honeybee venom manually extracted from venom gland (GV) and electrical stimulation (ESV) by 1-DE analysis**

|  | **Accession No.** | **Protein Name** | ***M*r (kDa)** | **p*I*** | **Origin** | **Score** | **matches** | **Unique** | **Sequence Coverage (%)** | **Gel Fraction (Figure S1）** | **E value** | **Mean emPAI±SE** | | **P-value** |
| --- | --- | --- | --- | --- | --- | --- | --- | --- | --- | --- | --- | --- | --- | --- |
|  |  |  |  |  |  |  |  |  |  |  |  | **ESV** | **GV** |  |
| toxins (16) | gi|28201825 | Melittin | 8.57 | 6.03 | ESV | 7983 | 194 | 2 | 18 | 1-12 |  | 2.88±0.73 ^a^ | 2.20±0.46 ^a^ | 0.160 |
|  |  |  |  |  | GV | 1184 | 53 | 1 | 19 | 1,6,8-10 | 4.00E-07 |  |  |  |
|  | gi\|5627 | Phospholipase A-2 | 19.05 | 7.18 | ESV | 13379 | 394 | 10 | 37 | 1-12 |  | 45.99±2.70 ^a^ | 47.35±0.35 ^a^ | 0.642 |
|  |  |  |  |  | GV | 12164 | 385 | 12 | 50 | 1,6-12 |  |  |  |  |
|  | gi\|187281543 | Venom dipeptidylpeptidase 4 precursor(Api m 5) | 88.34 | 5.72 | ESV | 325 | 15 | 10 | 33 | 2 |  | 0.78±0.08^a^ | 0.34±0.05^b^ | 0.013 |
|  |  |  |  |  | GV | 93 | 4 | 4 | 10 | 2 |  |  |  |  |
|  | gi\|67010041 | Major royal jelly protein 9 precursor(MRJP9) | 48.94 | 8.7 | ESV | 519 | 16 | 11 | 15 | 3,4 |  | 1.45±0.13 ^a^ | 0.61±0.06 ^b^ | 0.005 |
|  |  |  |  |  | GV | 609 | 28 | 5 | 16 | 4 |  |  |  |  |
|  | gi\|58585070 | Major royal jelly protein 8 precursor(MRJP8) | 47.33 | 6.00 | ESV | 525 | 18 | 10 | 19 | 3,4 |  | 1.03±0.07 ^a^ | 0.18±0.02 ^b^ | 0.000 |
|  |  |  |  |  | GV | 67 | 2 | 2 | 8 | 4 |  |  |  |  |
|  | gi\|187281550 | Venom carboxylesterase-6 precursor(Api m 8) | 63.94 | 9.33 | ESV | 224 | 6 | 5 | 19 | 3 |  | 0.40±0.04 ^b^ | 0.56±0.02 ^a^ | 0.025 |
|  |  |  |  |  | GV | 2975 | 99 | 9 | 20 | 3 |  |  |  |  |
|  | gi\|66821891 | Venom allergen acid phosphatase(Api m 3) | 44.11 | 5.63 | ESV | 1957 | 44 | 9 | 67 | 4 |  | 6.43±0.52 ^a^ | 2.43±0.41 ^b^ | 0.004 |
|  |  |  |  |  | GV | 1515 | 50 | 6 | 47 | 4 |  |  |  |  |
|  | gi\|58585182 | Hyaluronidase precursor | 44.46 | 8.67 | ESV | 1517 | 45 | 14 | 65 | 4,5 |  | 14.87±1.59 ^a^ | 2.50±0.08 ^b^ | 0.002 |
|  |  |  |  |  | GV | 925 | 43 | 4 | 38 | 4 |  |  |  |  |
|  | gi\|60115688 | Icarapin-like precursor | 24.83 | 4.51 | ESV | 634 | 18 | 4 | 21 | 5,6 |  | 1.15±0.08 ^a^ | 0.76±0.07 ^b^ | 0.026 |
|  |  |  |  |  | GV | 81 | 4 | 3 | 22 | 5,6 |  |  |  |  |
|  | gi\|110758297 | Phospholipase A2-like(PLA2-like) | 21.15 | 8.81 | ESV | 41 | 6 | 3 | 17 | 9 |  | 0.53±0.12 ^b^ | 1.22±0.10 ^a^ | 0.012 |
|  |  |  |  |  | GV | 68 | 2 | 2 | 21 | 10 |  |  |  |  |
|  | gi\|94400907 | Allergen Api m 6 precursor(Api m 6) | 10.38 | 9.83 | ESV | 259 | 11 | 7 | 47 | 11,12 |  | 12.12±1.25 ^a^ | 3.92±0.96 ^b^ | 0.007 |
|  |  |  |  |  | GV | 78 | 5 | 5 | 28 | 12 |  |  |  |  |
|  | gi\|58585166 | Apamin preproprotein | 5.51 | 8.77 | ESV | 132 | 4 | 1 | 19 | 12 | 1.00E-05 | 0.62±0 ^a^ | 0.62±0 ^a^ | 1 |
|  |  |  |  |  | GV | 54 | 1 | 1 | 19 | 12 | 5.10E-04 |  |  |  |
|  | gi|226533687 | Venom serine carboxypeptidase precursor(Api m 9) | 53.78 | 6.65 | GV | 147 | 6 | 1 | 4 | 3 | 3.60E-04 | 0 ^b^ | 0.14±0.02 ^a^ | 0 |
|  | gi|58585116 | Venom serine protease 34 precursor(Api m 7) | 46.40 | 8.64 | GV | 48 | 1 | 1 | 4 | 10 | 7.00E-05 | 0 ^b^ | 0.12±0.02 ^a^ | 0 |
|  | gi|58585104 | Vitellogenin | 202.12 | 6.29 | GV | 516 | 18 | 9 | 34 | 1 |  | 0 ^b^ | 0.54±0.18 ^a^ | 0 |
|  | gi|1708948 | Mast cell degranulating peptide(MCDP) | 6.062 | 9.87 | ESV | 74 | 2 | 1 | 18 | 12 | 1.10E-04 | 1.44±0 ^a^ | 0 ^b^ | 0 |
| Non-toxins (6) | gi|328780884 | Apolipophorins isofor*m* 1 | 202.12 | 8.65 | GV | 396 | 19 | 3 | 24 | 1 |  | 0 ^b^ | 0.33±0.08 ^a^ | 0 |
|  | gi|328780886 | Apolipophorins-like | 85.16 | 8.80 | GV | 72 | 2 | 2 | 12 | 2 |  | 0 ^b^ | 0.25±0.06 ^a^ | 0 |
|  | gi|149939403 | Hexamerin | 81.55 | 6.43 | GV | 54 | 3 | 3 | 6 | 2 |  | 0 ^b^ | 0.31±0.07 ^a^ | 0 |
|  | gi|335892796 | Peptidyl-prolyl cis-trans isomerase B precursor | 22.74 | 8.42 | GV | 50 | 2 | 2 | 11 | 11 |  | 0 ^b^ | 0.24±0.05 ^a^ | 0 |
|  | gi|328779578 | Lysozyme c-1 | 18.42 | 8.74 | GV | 222 | 4 | 1 | 10 | 11 | 8.90E-11 | 0 ^b^ | 0.24±0.07 ^a^ | 0 |
|  | gi|328783193 | Dehydrogenase/reductase SDR family member 11-like(SDR) | 28.19 | 8.44 | GV | 89 | 2 | 1 | 5 | 7 | 2.30E-06 | 0 ^b^ | 0.21±0.02 ^a^ | 0 |

All proteins are identified as *Apis mellifera* origin. Accession number is the unique number given to mark the entry of a protein in the database of NCBInr that used to search against in Mascot software. Theoretical molecular weight (*M*r), isoelectric point (p*I*) and score are search against from the database of NCBInr.. Matches are total peptide number assigned to the proteins.Unique is the number that exists only in one protein of a proteome. Sequence coverage is the ratio of the number of amino acids in peptides that yield by an experimental mass spectrum divided by the total number of amino acids in the protein sequence. Gel-fraction indicates from which fraction of the gel (Figure 1) the protein is identified. E-value is the theoretical probability of obtaining false-positive protein identification, it is reported only protein identified on the basis of one peptide and reach the threshold less than 1 in 1000. emPAI is a label free quantification of protein abundance calculated by Mascot software based on reference 30 and 31. Origin shows the proteins identified from the honeybee venom manually extracted from venom gland (GV) or electrical stimulation (ESV). The same up-case letter represents no statistical difference in abundance, “a” is statistical significant higher than “b”.
