# Supplementary material for: Proteome and phosphoproteome analysis of honeybee (Apis mellifera) venom collected from electrical stimulation and manual extraction of the venom gland
Source: BMC Genomics. 2013 Nov 7;14:766. doi: 10.1186/1471-2164-14-766 (PMC3835400; doi:10.1186/1471-2164-14-766)
Supplement: Additional file 3: Figure S2 — Quantitative comparisons of differential abundant proteins in honeybee (A. m. ligustica) venom manually collected from venom glands (GV) and electrical stimulated (ESV). The ratio of the protein abundance is ESV to GV. The positive values indicate higher protein abundance in ESV, negative values denote higher protein abundance in GV. The ratio is limited to 10, and error bar is standard deviation. Panel A, B and C are comparison of protein abundance analyzed by one-dimensional gel electrophoresis (1-DE), two-dimensional gel electrophoresis (2-DE) and shotgun analysis, respectively. [file 1471-2164-14-766-S3.doc]

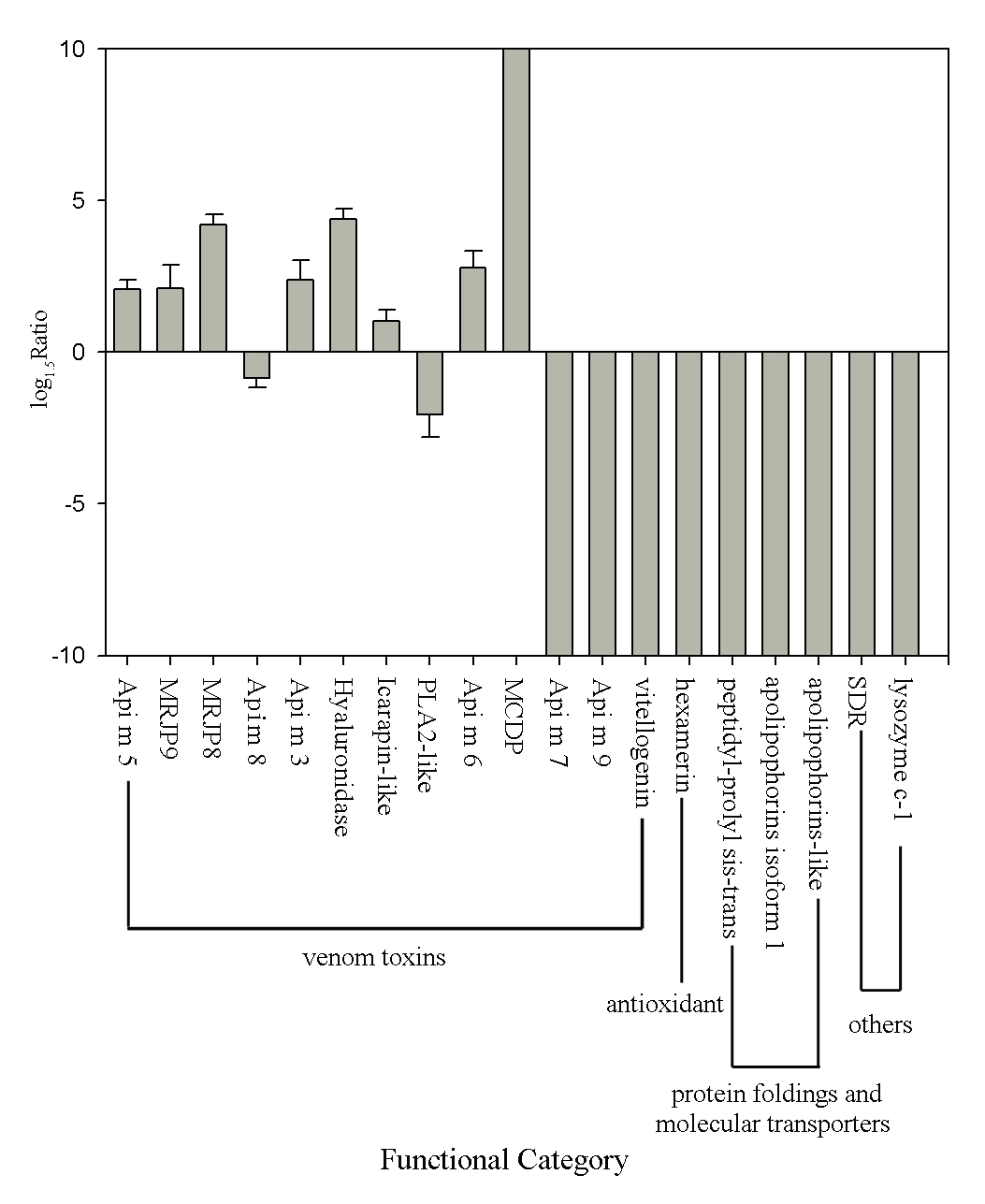


**A**


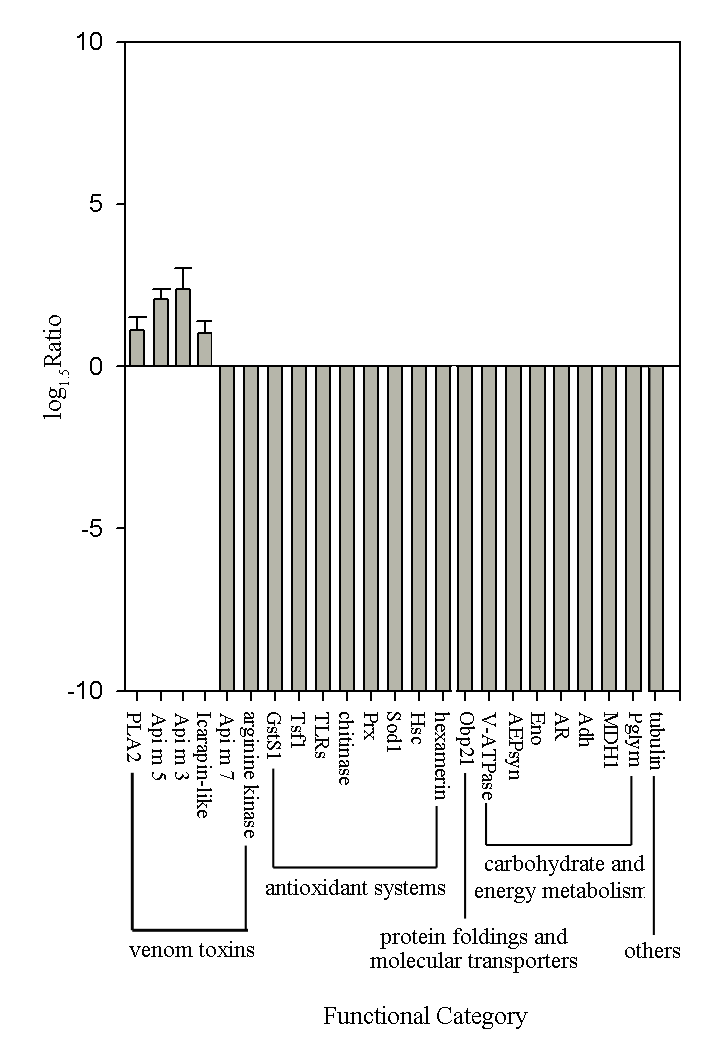


**B**


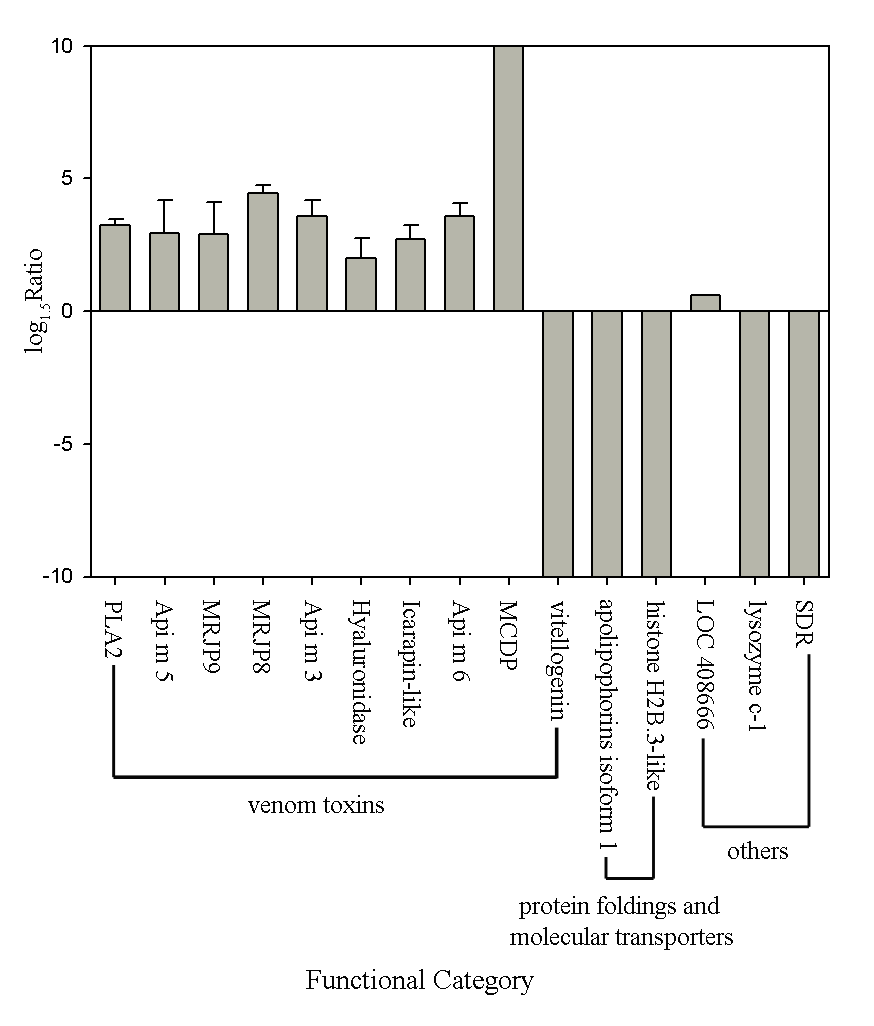


**C**

**Additional file 3: Figure S2.** Quantitative comparisons of differential abundant proteins in honeybee (*A. m. ligustica)* venom manually collected from venom glands (GV) and electrical stimulated (ESV). The ratio of the protein abundance is ESV to GV. The positive values indicate higher protein abundance in ESV, negative values denote higher protein abundance in GV. The ratio is limited to 10, and error bar is standard deviation. Panel A, B and C are comparison of protein abundance analyzed by one-dimensional gel electrophoresis (1-DE), two-dimensional gel electrophoresis (2-DE) and shotgun analysis, respectively.
