# Supplementary material for: Proteome and phosphoproteome analysis of honeybee (Apis mellifera) venom collected from electrical stimulation and manual extraction of the venom gland
Source: BMC Genomics. 2013 Nov 7;14:766. doi: 10.1186/1471-2164-14-766 (PMC3835400; doi:10.1186/1471-2164-14-766)
Supplement: Additional file 4: Figure S3 — Separation of honeybee (A. m. ligustica) venoms manually extracted from the venom gland (GV) and electrical stimulation (ESV) using two-dimensional gel electrophoresis (2-DE). 500 μg of each sample are subjected to 2-DE and the proteins are stained using a mass spectrometry compatible silver-staining method. Number-labeled spots are cut out and subjected to tryptic digestion for mass spectrometry analysis. [file 1471-2164-14-766-S4.doc]

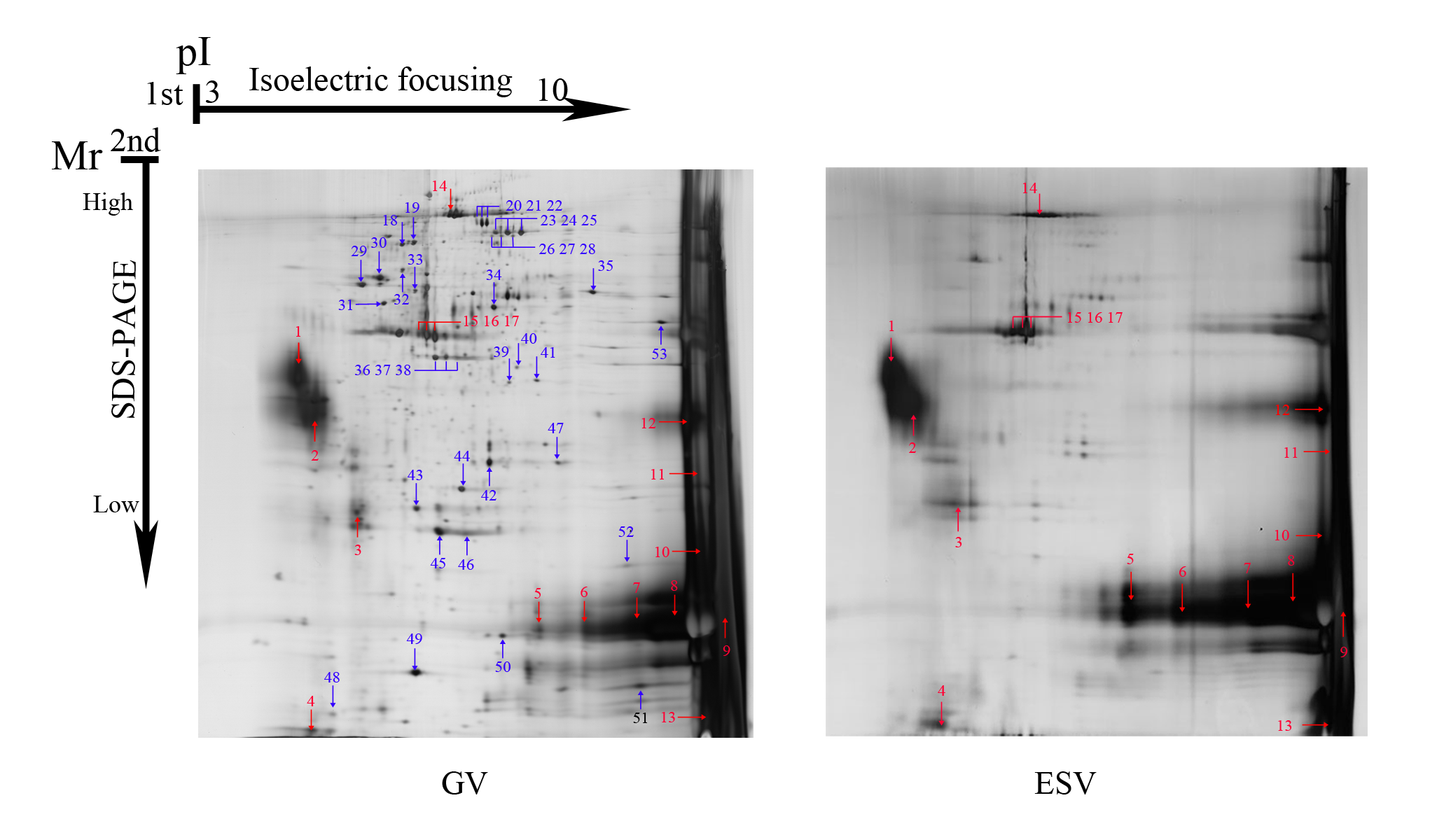


**Additional file 4: Figure S3.** Separation of honeybee (*A. m. ligustica*) venoms manually extracted from the venom gland (GV) and electrical stimulation (ESV) using two-dimensional gel electrophoresis (2-DE). 500 μg of each sample are subjected to 2-DE and the proteins are stained using a mass spectrometry compatible silver-staining method. Number-labeled spots are cut out and subjected to tryptic digestion for mass spectrometry analysis.
