# Supplementary material for: Proteome and phosphoproteome analysis of honeybee (Apis mellifera) venom collected from electrical stimulation and manual extraction of the venom gland
Source: BMC Genomics. 2013 Nov 7;14:766. doi: 10.1186/1471-2164-14-766 (PMC3835400; doi:10.1186/1471-2164-14-766)
Supplement: Additional file 5: Table S2 — Identification of different abundant proteins between honeybee venom manually extracted from venom glands (GV) and electrical stimulation (ESV) by 2-DE analysis. [file 1471-2164-14-766-S5.doc]

**Additional file 5: Table S2. Identification of different abundant proteins between honeybee venom manually extracted from venom glands (GV) and electrical stimulation (ESV) by 2-DE analysis**

|  | **Accession No.** | **Protein Name** | ***M*r(kDa)** | **p*I*** | **Spot No.** | **Score** | **matches** | **Unique** | **Sequence Coverage (%)** | **Protein abundance** | | **P-value** |
| --- | --- | --- | --- | --- | --- | --- | --- | --- | --- | --- | --- | --- |
|  |  |  |  |  |  |  |  |  |  | **ESV** | **GV** |  |
| toxins (4) | gi|187281543 | Venom dipeptidyl peptidase IV precursor | 88.34 | 5.72 | 14 | 366 | 12 | 8 | 13 | 7.47±3.79E-02 ^a^ | 7.16±3.14E-02 ^b^ | 1.54E-06 |
|  | gi\|66821891 | Venom allergen acid phosphatase | 44.11 | 5.63 | 15 | 87 | 3 | 2 | 10 | 7.21±0.12 ^a^ | 6.9±7.58E-002 ^b^ | 6.93E-04 |
|  |  |  |  |  | 16 | 1332 | 37 | 9 | 60 | 7.9±0.08 ^a^ | 7.12±0.11 ^b^ | 1.07E-04 |
|  |  |  |  |  | 17 | 958 | 27 | 8 | 51 | 7.6±0.09 ^a^ | 7.45±4.8E-002 ^b^ | 0.01 |
|  | gi\|60115688 | Icarapin-like precursor | 24.83 | 4.51 | 1 | 241 | 7 | 6 | 25 | 8.49±8.17E-02 ^a^ | 8.09±9.96E-02 ^b^ | 2.14E-04 |
|  |  |  |  |  | 2 | 226 | 9 | 3 | 10 | 9.07±0.1 ^a^ | 8.78±7.90E-002 ^b^ | 5.13E-04 |
|  |  |  |  |  | 4 | 116 | 3 | 2 | 10 | 6.11±0.05 ^a^ | 5.99±0.18 ^b^ | 0.02 |
|  | gi\|5627 | Phospholipase A-2 | 19.05 | 7.18 | 5 | 322 | 11 | 7 | 44 | 8.93±0.13 ^a^ | 8.44±0.06 ^b^ | 0.008 |
|  |  |  |  |  | 6 | 405 | 14 | 8 | 53 | 9.01±0.1 ^a^ | 8.56±8.30E-002 ^b^ | 5.41E-05 |
|  |  |  |  |  | 7 | 681 | 22 | 9 | 60 | 9.12±8.46E-002 ^a^ | 8.73±0.11 ^b^ | 1.54E-04 |
|  |  |  |  |  | 8 | 1379 | 35 | 11 | 62 | 9±0.12 ^a^ | 8.68±4.14E-002 ^b^ | 2.69E-04 |
|  |  |  |  |  | 9 | 553 | 14 | 7 | 48 | 8.81±0.12 ^a^ | 8.47±0.22 ^b^ | 0.006 |
|  |  |  |  |  | 10 | 575 | 20 | 10 | 62 | 7.68±0.28 ^a^ | 7.07±0.23 ^b^ | 0.002 |
|  |  |  |  |  | 11 | 890 | 24 | 11 | 71 | 8.13±0.18 | 7.63±0.3 ^b^ | 0.005 |
|  |  |  |  |  | 12 | 163 | 5 | 4 | 53 | 6.78±0.28 ^a^ | 6.29±0.35 ^b^ | 0.01 |
|  |  |  |  |  | 13 | 439 | 14 | 7 | 62 | 7.85±8.2E-002 ^a^ | 7.48±0.24 ^b^ | 0.004 |
|  |  |  |  |  | 51 | 726 | 19 | 8 | 53 | 6.38±0.23 ^b^ | 6.69±0.23 ^a^ | 0.03 |
|  |  |  |  |  | 52 | 85 | 2 | 2 | 13 | 6.46±0.24 ^b^ | 7.17±0.19 ^a^ | 4.64E-04 |
|  |  |  |  |  | 53 | 192 | 7 | 6 | 37 | 6.6±0.41 ^b^ | 7.16±0.43 ^a^ | 0.03 |
| non-toxins (20) | gi|328789531 | Hypothetical protein LOC408666 | 37.2 | 7.3 | 3 | 77 | 2 | 2 | 22 | 8.83±0.11 ^a^ | 8.5±9.83E-002 ^b^ | 4.36E-04 |
|  |  |  |  |  | 45 | 181 | 6 | 5 | 27 | 7.55±3.65E-002 ^b^ | 8.07±2.93E-002 ^a^ | 5.22E-08 |
|  |  |  |  |  | 46 | 67 | 3 | 2 | 11 | 7.26±0.16 ^b^ | 7.98±0.11 ^a^ | 2.81E-05 |
|  | gi\|66515272 | V-type proton ATPase catalytic subunit A-like isoform 1 | 68.61 | 5.3 | 18 | 732 | 22 | 7 | 41 | 0 ^b^ | 7.05±8.61E-02 ^a^ | 0 |
|  |  |  |  |  | 33 | 69 | 2 | 2 | 5 | 0 ^b^ | 7.15±0.31 ^a^ | 0 |
|  | gi\|22982210 | Heat shock protein cognate 4 | 71.83 | 5.43 | 19 | 651 | 25 | 20 | 38 | 0 ^b^ | 7.22±8.81E-002 ^a^ | 0 |
|  |  |  |  |  | 32 | 86 | 5 | 5 | 9 | 0 ^b^ | 7.03±0.11 ^a^ | 0 |
|  | gi\|110755367 | Toll-like receptor 13-like isoform 1 | 76.63 | 6.09 | 21 | 419 | 14 | 9 | 18 | 0 ^b^ | 7.55±0.14 ^a^ | 0 |
|  |  |  |  |  | 22 | 277 | 8 | 7 | 14 | 0 ^b^ | 7.25±9.70E-02 ^a^ | 0 |
|  | gi\|58585086 | Transferrin 1 precursor | 80.03 | 6.77 | 23 | 456 | 13 | 11 | 20 | 0 ^b^ | 7.25±9.7E-02 ^a^ | 0 |
|  |  |  |  |  | 24 | 1138 | 32 | 26 | 50 | 0 ^b^ | 7.55±0.14 ^a^ | 0 |
|  |  |  |  |  | 25 | 1263 | 32 | 28 | 53 | 0 ^b^ | 7.54±0.11 ^a^ | 0 |
|  | gi\|149939403 | Hexamerin | 81.55 | 6.43 | 26 | 82 | 4 | 3 | 2 | 0 ^b^ | 6.75±0.16 ^a^ | 0 |
|  |  |  |  |  | 27 | 48 | 2 | 2 | 2 | 0 ^b^ | 6.28±0.16 ^a^ | 0 |
|  |  |  |  |  | 28 | 178 | 4 | 4 | 6 | 0 ^b^ | 6.75±0.16 ^a^ | 0 |
|  | gi\|48095525 | Tubulin beta-1 chain | 50.6 | 4.75 | 29 | 1378 | 37 | 3 | 53 | 0 ^b^ | 7.69±0.13 ^a^ | 0 |
|  |  |  |  |  | 30 | 375 | 11 | 2 | 28 | 0 ^b^ | 7.55±4.38E-02 ^a^ | 0 |
|  | gi\|328785025 | ATP synthase subunit beta, mitochondrial | 55.32 | 5.3 | 31 | 458 | 15 | 6 | 24 | 0 ^b^ | 6.85±0.18 ^a^ | 0 |
|  | gi\|328776580 | Enolase-like | 39.28 | 6.24 | 34 | 344 | 6 | 4 | 20 | 0 ^b^ | 7.38±3.68E-002 ^a^ | 0 |
|  | gi\|66514614 | Chitinase-like protein Idgf4-like | 50 | 8.06 | 35 | 150 | 6 | 6 | 16 | 0 ^b^ | 7.49±0.16 ^a^ | 0 |
|  | gi\|58585146 | Arginine kinase | 40.33 | 5.66 | 36 | 230 | 8 | 7 | 28 | 0 ^b^ | 7.42±6.03E-02 ^a^ | 0 |
|  |  |  |  |  | 37 | 1269 | 33 | 4 | 60 | 0 ^b^ | 7.42±6.03E-02 ^a^ | 0 |
|  |  |  |  |  | 38 | 506 | 14 | 2 | 42 | 0 ^b^ | 7.49±0.12 ^a^ | 0 |
|  | gi\|66525576 | Aldose reductase-like | 36.46 | 6.26 | 39 | 139 | 4 | 4 | 16 | 0 ^b^ | 6.81±0.1 ^a^ | 0 |
|  | gi\|328780312 | Alcohol dehydrogenase [NADP+] A-like isoform 1 | 27.37 | 6.89 | 40 | 143 | 5 | 5 | 25 | 0 ^b^ | 6.86±9.09E-02 ^a^ | 0 |
|  | gi\|66506786 | Malate dehydrogenase cytoplasmic-like | 36.20 | 6.25 | 41 | 77 | 2 | 2 | 7 | 0 ^b^ | 6.98±0.2 ^a^ | 0 |
|  | gi\|58585116 | Venom serine protease 34 precursor | 46.40 | 8.64 | 42 | 412 | 13 | 8 | 27 | 0 ^b^ | 7.62 ±7.21E-002 ^a^ | 0 |
|  | gi\|380020933 | Glutathione S-transferase-like isoform 1 | 23 | 5.49 | 43 | 481 | 18 | 4 | 48 | 0 ^b^ | 7.64±0.14 ^a^ | 0 |
|  | gi\|283436152 | Peroxiredoxin-like protein | 25.23 | 5.88 | 44 | 368 | 16 | 8 | 38 | 0 ^b^ | 7.42±0.11 ^a^ | 0 |
|  | gi\|66550890 | Phosphoglycerate mutase 2-like | 35.40 | 9.36 | 47 | 112 | 3 | 3 | 9 | 0 ^b^ | 7.26±0.18 ^a^ | 0 |
|  | gi\|66535784 | Odorant binding protein 21 precursor | 15.54 | 4.76 | 48 | 122 | 4 | 3 | 27 | 0 ^b^ | 8.06±8.72E-02 ^a^ | 0 |
|  |  |  |  |  | 49 | 292 | 10 | 4 | 40 | 0 ^b^ | 8.06±8.72E-02 ^a^ | 0 |
|  | gi\|295849268 | Superoxide dismutase 1 | 15.8 | 6.21 | 50 | 75 | 2 | 2 | 26 | 0 ^b^ | 7.44±0.14 ^a^ | 0 |

All proteins are identified as *Apis mellifera* origin Spot numbers are marked in accordance with the corresponding numbers on Figure S3. Accession number is the unique number given to mark the entry of a protein in the database of NCBInr that used to search against in Mascot software. Theoretical molecular weight (*M*r), isoelectric point (p*I*) and score are search against from the database of NCBInr. Protein name is given according to the annotation in database. Matches are total peptide number assigned to the proteins. Unique is the number that exists only in one protein of a proteome. Sequence coverage is the ratio of the number of amino acids in peptides that yield by experimental mass spectra divided by the total number of amino acids in the protein sequence. Protein abundance is the mean of log normalized value of protein and standard deviation (Mean±SD). “a” donates significant higher abundance than “b”.
