# Supplementary material for: Proteome and phosphoproteome analysis of honeybee (Apis mellifera) venom collected from electrical stimulation and manual extraction of the venom gland
Source: BMC Genomics. 2013 Nov 7;14:766. doi: 10.1186/1471-2164-14-766 (PMC3835400; doi:10.1186/1471-2164-14-766)
Supplement: Additional file 6: Table S3 — Identification and quantitation of protein in honeybee venom manually extracted from venom gland (GV) and electrical stimulation (ESV) by shotgun analysis. [file 1471-2164-14-766-S6.doc]

**Additional file 6: Table S3. Identification and quantitation of protein in honeybee venom manually extracted from venom gland (GV) and electrical stimulation (ESV) by shotgun analysis**

|  | **Accession No.** | **Protein Name** | ***M*r (kDa)** | **p*I*** | **Origin** | **Score** | **Matches** | **Unique** | **Sequence Coverage (%)** | **E value** | **Mean emPAI±SE** | | **P-value** |
| --- | --- | --- | --- | --- | --- | --- | --- | --- | --- | --- | --- | --- | --- |
|  |  |  |  |  |  |  |  |  |  |  | **ESV** | **GV** |  |
| toxins  (13) | gi|28201825 | Melittin | 8.57 | 6.03 | ESV | 3291 | 165 | 2 | 19 |  | 2.75±0^a^ | 2.75±0^a^ | 1 |
|  |  |  |  |  | GV | 4217 | 256 | 2 | 19 |  |  |  |  |
|  | gi\|5627 | Phospholipase A-2(PLA2) | 19.05 | 7.18 | ESV | 8941 | 284 | 15 | 79 |  | 156.40±26.99^a^ | 41.66±6.05^b^ | 0.014 |
|  |  |  |  |  | GV | 12043 | 493 | 11 | 70 |  |  |  |  |
|  | gi\|187281543 | Venom dipeptidylpeptidase 4 precursor(Api m 5) | 88.34 | 5.72 | ESV | 483 | 20 | 6 | 31 |  | 0.78±0.05^a^ | 0.23±0.06^b^ | 0.003 |
|  |  |  |  |  | GV | 287 | 8 | 1 | 19 | 1.10E-06 |  |  |  |
|  | gi\|67010041 | Major royal jelly protein 9 precursor(MRJP9) | 48.94 | 8.7 | ESV | 1103 | 36 | 10 | 52 |  | 1.68±0.30 ^a^ | 0.51±0.23 ^b^ | 0.038 |
|  |  |  |  |  | GV | 150 | 4 | 2 | 43 |  |  |  |  |
|  | gi\|58585070 | Major royal jelly protein 8 precursor(MRJP8) | 47.33 | 6.00 | ESV | 838 | 34 | 10 | 32 |  | 1.43±0.27 ^a^ | 0.23±0.02 ^b^ | 0.012 |
|  |  |  |  |  | GV | 78 | 2 | 2 | 9 |  |  |  |  |
|  | gi\|66821891 | Venom allergen acid phosphatase(Api m 3) | 44.11 | 5.63 | ESV | 2603 | 78 | 12 | 62 |  | 6.20±1.01 ^a^ | 1.45±0.09 ^b^ | 0.010 |
|  |  |  |  |  | GV | 1235 | 38 | 8 | 36 |  |  |  |  |
|  | gi\|58585182 | Hyaluronidase precursor | 44.46 | 8.67 | ESV | 1780 | 50 | 6 | 57 |  | 5.14±0.81 ^a^ | 2.29±0.25 ^b^ | 0.029 |
|  |  |  |  |  | GV | 976 | 30 | 8 | 40 |  |  |  |  |
|  | gi\|60115688 | Icarapin-like precursor | 24.83 | 4.51 | ESV | 468 | 19 | 6 | 15 |  | 1.33±0.16 ^a^ | 0.44±0 ^b^ | 0.005 |
|  |  |  |  |  | GV | 83 | 3 | 2 | 11 |  |  |  |  |
|  | gi\|110758297 | Phospholipase A2-like(PLA2-like) | 21.15 | 8.81 | ESV | 84 | 3 | 3 | 28 |  | 1.92±0.6 ^a^ | 1.03±0.16 ^a^ | 0.229 |
|  |  |  |  |  | GV | 46 | 2 | 2 | 28 |  |  |  |  |
|  | gi\|94400907 | Allergen Api m 6 precursor(Api m 6) | 10.38 | 9.83 | ESV | 266 | 14 | 4 | 47 |  | 9.92±0.95 ^a^ | 2.31±0.31 ^b^ | 0.002 |
|  |  |  |  |  | GV | 74 | 11 | 3 | 38 |  |  |  |  |
|  | gi\|223850 | Secapin | 2.98 | 9.84 | ESV | 319 | 15 | 1 | 32 | 3.00E-04 | 2.12±0.9 ^a^ | 1.22±0 ^a^ | 0.374 |
|  |  |  |  |  | GV | 37 | 1 | 1 | 32 | 9.40E-04 |  |  |  |
|  | gi\|58585104 | Vitellogenin | 202.12 | 6.29 | GV | 88 | 3 | 3 | 8 |  | 0 ^b^ | 0.15±0.01 ^a^ | 0 |
|  | gi\|1708948 | Mast cell degranulating peptide(MCDP) | 6.062 | 9.87 | ESV | 442 | 22 | 1 | 18 | 1.20E-04 | 1.44±0 ^a^ | 0 ^b^ | 0 |
| non-toxins (7) | gi|328789531 | Hypothetical protein LOC408666 | 37.20 | 7.3 | ESV | 101 | 5 | 2 | 29 |  | 0.50±0 ^a^ | 0.39±0 ^b^ | 0.000 |
|  |  |  |  |  | GV | 439 | 15 | 2 | 8 |  |  |  |  |
|  | gi|328782499 | Proactivator polypeptide isoform 1 | 103.06 | 5.32 | ESV | 201 | 9 | 7 | 13 |  | 0.26±0.04 ^a^ | 0.23±0.06 ^a^ | 0.781 |
|  |  |  |  |  | GV | 692 | 18 | 5 | 21 |  |  |  |  |
|  | gi|328790510 | DnaJ homolog subfamily B member 11-like | 41.66 | 5.66 | ESV | 51 | 1 | 1 | 11 | 6.70E-04 | 0.13±0.02 ^a^ | 0.13±0.02 ^a^ | 1.000 |
|  |  |  |  |  | GV | 378 | 7 | 1 | 6 | 1.90E-08 |  |  |  |
|  | gi|328780884 | Apolipophorins isofor*m* 1 | 202.12 | 8.65 | GV | 58 | 1 | 1 | 4 | 6.60E-05 | 0 ^b^ | 0.05±0.01 ^a^ | 0 |
|  | gi|328779578 | Lysozyme c-1 | 18.42 | 8.74 | GV | 53 | 1 | 1 | 12 | 2.80E-04 | 0 ^b^ | 0.17±0 ^a^ | 0 |
|  | gi|328783193 | Dehydrogenase/reductase SDR family member 11-like(SDR) | 28.19 | 8.44 | GV | 55 | 2 | 2 | 21 |  | 0 ^b^ | 0.33±0.09 ^a^ | 0 |
|  | gi|110749558 | Histone H2B.3-like | 13.69 | 10.39 | GV | 92 | 2 | 1 | 12 | 2.40E-06 | 0 ^b^ | 0.24±0 ^a^ | 0 |

All proteins are identified as *Apis mellifera* origin. Accession number is the unique number given to mark the entry of a protein in the database of NCBInr that used to search against in Mascot software. Theoretical molecular weight (*M*r), isoelectric point (p*I*) and score are search against from the database of NCBInr.. Matches are total peptide number assigned to the proteins. Unique peptide is the number that exists only in one protein of a proteome. Sequence coverage is the ratio of the number of amino acids in peptides that yield by experimental mass spectra divided by the total number of amino acids in the protein sequence. E-value is the theoretical probability of obtaining false-positive protein identification, it is reported only protein identified on the basis of one peptide and reach the threshold less than 1 in 1000. emPAI is a label free quantification of protein abundance calculated by Mascot software based on reference 30 and 31. Origin shows the proteins identified from the honeybee venom manually extracted from venom gland (GV) or electrical stimulation (ESV). The same up-case letter represents no difference in abundance, and “a” is statistical significant higher than “b”.
